# EO-Performance relationships in Reverse Internationalization by Chinese Global Startup OEMs: Social Networks and Strategic Flexibility

**DOI:** 10.1371/journal.pone.0162175

**Published:** 2016-09-15

**Authors:** Tachia Chin, Sang-Bing Tsai, Kai Fang, Wenzhong Zhu, Dongjin Yang, Ren-huai Liu, Richard Ting Chang Tsuei

**Affiliations:** 1 School of Management, Hangzhou Dianzi University, Hangzhou, China; 2 College of Business Administration, Dongguan University of Technology, Guangdong, China; 3 Zhongshan Institute, University of Electronic Science and Technology of China. Guangdong, China; 4 Law School, Nankai University, Tianjin, China; 5 The Fu Foundation School of Engineering and Applied Science, Columbia University in The City of New York, New York, United States of America; 6 School of Business, Guangdong University of Foreign Studies, Guangzhou, China; 7 School of Management, Jinan University, Guangzhou, China; 8 Research Center of Strategic Management, Jinan University, Guangzhou, China; 9 Institute of Innovation and Development, Hangzhou Dianzi University, Hangzhou, China; 10 Guanxi Wuzhou Zhongheng Group Co. Ltd, Wuzhou, Guangxi, China; Utrecht University, NETHERLANDS

## Abstract

Due to the context-sensitive nature of entrepreneurial orientation (EO), it is imperative to in-depth explore the EO-performance mechanism in China at its critical, specific stage of economic reform. Under the context of “reverse internationalization” by Chinese global startup original equipment manufacturers (OEMs), this paper aims to manifest the unique links and complicated interrelationships between the individual EO dimensions and firm performance. Using structural equation modeling, we found that during reverse internationalization, *proactiveness* is positively related to performance; *risk taking* is not statistically associated with performance; *innovativeness* is negatively related to performance. The proactiveness-performance relationship is mediated by Strategic flexibility and moderated by social networking relationships. The dynamic and complex institutional setting, coupled with the issues of overcapacity and rising labor cost in China may explain why our distinctive results occur. This research advances the understanding of how contingent factors (social network relationships and strategic flexibility) facilitate entrepreneurial firms to break down institutional barriers and reap the most from EO. It brings new insights into how Chinese global startup OEMs draw on EO to undertake *reverse internationalization*, responding the calls for unraveling the heterogeneous characteristics of EO sub-dimensions and for more contextually-embedded treatment of EO-performance associations.

## Introduction

Entrepreneurial orientation (EO), as the strategy making process characterizing a firm's proclivity toward entrepreneurship, is generally recognized as a significant driver of firm performance, especially for enterprises operating in rapidly changing and competitive environment [1–2]. However, the effect of EO on performance is not always positive and linear, but contingency-oriented and context-specific [3–5]. Specifically, the EO-performance relationship won’t remain static; the impact of EO on firm performance may differ if in various industrial settings, development stages, or under market turbulence and financial crisis [6–8]. Hence, despite the increasing attention, greater insights into the EO- performance linkages in China is still largely required, given this nation, as the world’s biggest emerging market, is at a critical period of its economic and political reforms in developing a unique socialistic market system with the coexistence of socialist and market-based capitalist characteristics. While the world economy has remained sluggish since the 2009 global financial crisis and is expected to continue slowing down after the sudden hit by Brexit in 2016, it is vital to investigate in depth how small, domestic entrepreneurial firms in China choose to react to worldwide challenges. To fill this gap, this paper seeks to elucidate the EO-performance mechanisms in China from a wider and contingency perspective.

Since China’s reform and opening-up in the late 90’s, an increasing number of foreign direct investments (FDIs) have been flowing into China for benefiting from its low-cost production and labor. Many Chinese domestic firms have thus taken this opportunity to serve as original equipment manufacturers (OEMs) for foreign entrants, and rapidly internationalize themselves from inception by export at home without needing to bear higher risks of directly doing business overseas. Along with this trend, more and more export-driven global startup OEMs have come into existence, becoming a prevalent and typical type of entrepreneurial firms in China for the past three decades [9]. Evidence also manifests that these global startup OEMs that compete abroad at early age have learned a variety of advanced knowledge and technology through collaborating with FDIs, whereby some ambitious ones even further upgraded to engage in original brand manufacturing (OBM) [10–11].

However, in recent years, facing profound global economic uncertainty, Chinese OEMs could no longer rely on export to well-regulated mature markets as in the past but seek opportunities in their vast, yet disordered home markets where the consumer demand is growing despite unsound legal and economic institutions. According to Chin et al. [9], this distinctive, ongoing entrepreneurial phenomenon in China’s manufacturing sector can be defined as *“reverse internationalization”*- the prominent *direction changes* about the growth and learning trajectories of Chinese entrepreneurial OEMs responding to the new fundamental transformation of global economy. The *“reverse internationalization” by global startups OEMs* reflects the intricate connections between the world’s economic problems and China’s industrial transition, thus providing a compelling strategic background for us to probe into the context-specific EO-performance relationship with a much broader perspective. Moreover, with the aim to advance our understanding of the EO effects, we refer to the claims of previous research [8], considering EO as a multidimensional phenomenon (i.e., innovativeness, proactiveness and risk-taking) here.

Scholars argue that more attention should be paid to identify pivotal context-specific factors that may moderate the EO-performance relationship because in prior research, the contingency-oriented nature of the EO-performance linkage has resulted in inconsistent findings under different circumstances, especially when involving with contextual moderators [12]. Hence, this paper focuses on two critical contingent variables: social networking and strategic flexibility that embody the unique institutional idiosyncrasies in China. Despite that the effects of social networks as a critical social capital on entrepreneurial firms’ performance have been widely identified [13–15], whether social networking (also called *Guanxi*, an indigenous appellation in China) moderates the EO-performance relationships under the context of *reverse internationalization* remains an unexplored area. Krasus et al. [1] indicates that facing a complex institutional environment like China, a firm’s EO might synthesize a certain level of *strategic flexibility* that enhances the flexible use of its resources to overcome institutional constraints. Considering the foregoing, we aim to investigate how *Guanxi* and strategic flexibility intervene in the EO-performance mechanisms in *reverse internationalization* here.

In sum, this study makes several contributions to the literature. First and foremost, we elucidate the unique EO-performance mechanisms in a particular context, *reverse internationalization* by Chinese global startup OEMs, thus characterizing the context-sensitive nature of EO [6]. Second, echoing the recent calls from scholars [16], we analyze the impact of individual EO dimensions on performance. Third, by identifying the moderator of *guanxi* and the mediator of strategic flexibility on the EO-performance linkages, we highlight the importance of contingent variables in steering the paths where the individual dimensions of EO may directly enhance or be indirectly translated into firm performance in China.

## Theoretical Foundation and Hypotheses

### The conceptualization and dimensionality of EO

Despite the ongoing debates on whether EO should be defined as a disposition or behavior construct or measured as a reflective or formative construct [17], most scholars agree with Miller’s [18] definition that EO as a firm’s strategic posture of specific processes, practices and activities is constituted by three idiosyncratic components—innovativeness, risk taking, and proactiveness—which enable firms to create value by engaging in product-market innovation, undertaking somewhat risky ventures, and acting on future demands [19–20]. Conceptually, **innovativeness** represents a firm's strong commitment to embrace creative ideas, novel technology and products; **risk taking** refers to a firm's willingness to make large resource commitments with a reasonable chance of costly failures; **proactiveness** embodies a firm's opportunity-seeking tendency such as entering new markets and introducing new products and services ahead of the competition in anticipation of the future market trends [1, 21].

Some scholars suggest that the above-mentioned three characteristics of EO should be viewed as a joint/ integral construct consisting of three sub-dimensions that must positively covary in order for an EO to be exhibited [3, 22]. This conceptualization posits that the three attributes of EO, acting as a unidimensional gestalt, should be aggregated together when measuring EO. However, a recent view seeks to uncover the unique roles played by each of the EO dimensions, clarifying the differential effects of individual EO factors on firm-level outcomes [19, 23]. This multi-dimensional standpoint underscores the potentially distinct nature of the three EO dimensions and their corresponding unique contributions to firm performance. Research has indicated the significance of assessing the impact of individual EO dimensions on outcome variables, especially about examining the performance of small-to-medium sized enterprises (SMEs) [16, 23]. Given the possession of constrained resources, each sub-dimension of EO may mirror a distinctive strategic posture by a SME in determining the level and distribution of resource commitment with its unique cost-benefit trade-offs. Whereas our focus on a specific type of SMEs here, we follow this logic and expect that the individual dimensions of EO may exhibit heterogeneous effects on a global startup OEM’s performance under the context of *reverse internationalization*.

### EO-performance relationships in the context of “reverse internationalization”

The EO-performance relationships have been found to be very discordant, complex, and particularly context-sensitive [6,8,16]. By adopting a uni-dimensional EO construct, many studies suggest a positive, linear effect of EO on performance [3]; however, some research analyzing Chinese data shows that EO may not always translate into improved performance above a saturation point but rather has a curvilinear relationship with performance [22]. While taking the dimensionality of EO into consideration, research presents even more discrepant results. Kraus et al. [1] demonstrates that during the turbulent times, risk-taking is negatively related to performance, innovativeness has no impact, but proactiveness still contributes to performance; Kollmann and Stockmann [20] discover a negative association between risk-taking and performance despite innovative- and proactiveneess-performance relationships remain positive. In contrast, Rauch et al. [2] manifest a positive association between risk-taking and performance while some scholars even report the presence of non-linearity in EO-performance relationships [16]. Given the inconsistent findings, Dai et al. [23] claim that the EO-performance relationships may differ depending on whether the EO construct is presumed to be uni- or multi-dimensional. The evidence above corroborates the eminent contingent and somewhat emic nature of EO with its diversified impact on firm outcomes. Moreover, it implicitly sheds light on the imperative to further investigate the distinct influences of individual dimensions of EO on performance in a specific, representative entrepreneurial environment.

The *reverse internationalization by Chinese global startup OEMs*, as characterized by Chin et al. [9] is just a peculiar, entrepreneurial, and increasingly prevalent phenomenon in China that is very suitable for our research setting. In the past three decades, numerous FDIs have entered China for capitalizing on its low-cost production, which actually facilitate the emergence of Chinese global startup OEMs that embarked on their business by serving as contracting producers for international well-known brands (e.g., Nike, Gucci, & Apple) or large multinational manufacturers (e.g. Foxconn) [10–11]. The outsourcers that usually came from more developed economies might frequently transfer a certain level of technology know-how to the OEM suppliers, in order to ensure the quality of their end products. Hence, the acquisition of manufacturing, technological, and marketing knowledge has been widely recognized as the most valuable benefit obtained by Chinese OEMs through cooperating with foreign clients/buyers at home [9, 11].

However, the consecutive 2009 financial crisis and 2010 Europe debt crisis resulted in global recession and China’s export stagnation, which has further triggered the strategic trend of *reverse internationalization* by Chinese global startup OEMs. It is because many of such firms have been continuing to lose low-cost advantages in international competitions since then [24, 25], and thus have targeted China’s tremendous market to offset their loss in export. As a matter of fact, the strategic option of “reverse internationalization” by Chinese global startup OEMs is also partly encouraged by the Chinese government according to the *China’s 12*^*th*^ *and 13*^*th*^ *Five-Year Economic Plans (2011–2015 & 2016–2020)* [9]. This ‘reverse internationalization’, in this sense, characterizes a new growth strategy by Chinese OEMs to venture back to cultivate their domestic market, as a result of choosing between the escalating uncertainty and precipitous profit decline in mature markets and the growing demand at home.

One of the severest challenges China confronts is to incorporate two antagonistic ideologies-the socialist and the capitalist structures-into a unified institutional system. This could impose a variety of stress and confusion, and even generate unexpected institutional frictions and constraints that may diminish the value of EO for such global startup OEMs [10, 24]. In this vein, despite huge potential for growth, the Chinese market appears to be very dynamic and complex [24, 25]; the global startup OEMs, therefore, still have to change their learning strategies and patterns from learning from developed-country partners to learning from the incumbents in domestic markets. Viewed from this angle, this ‘reverse internationalization’ embodies the unique *direction changes* on the growth and learning trajectories of Chinese entrepreneurial OEMs in an attempt to survive a fast-changing and fiercely competitive situation. Taking the aforementioned discussions together, ‘reverse internationalization’ is particularly appropriate for assessing the impact of the broader conceptualization of EO on firm performance during China’s socio economic reform.

### Innovativeness and performance

Innovativeness is often regarded as a critical factor in facilitating growth by introducing and investing in new products, services, and cutting-edge technologies with high profit potential or market value [17]. This EO dimension generally involves a higher level of R & D investment and a large prior expenditure of organizational resources, which tends to provide long-term rather than short-term returns [16, 23].

Chinese global startup OEMs used to confine their business activities to labor-intensive production, relying on China’s huge labor endowment and low-wage advantages to compete in export markets [25]. Compared with large and foreign-invested manufacturers, such entrepreneurial OEMs had been confronted more institution-based barriers in the area of innovation in China, such as limited access to financial support and weak abilities to protect their intellectual property rights [24, 26]. Research also indicates that it seems to be quite difficult for small-and medium- sized OEMs to harvest from innovations than they deserve in China [22, 26]. Hence, global startup OEMs, without a legacy of innovation, usually invest very little in R & D.

As noted earlier, the 2009 and 2010 global economic crises triggered the reverse internationalization phenomenon in China. Due to an appreciating currency, rising minimum wage, tighter environmental protection policy, and severer competitions from other developing countries, Chinese global startup OEMs that have been facing sharp drop in export volume are endeavoring to benefit from their domestic markets with more promising opportunities [9, 24]. At this critical time, the strategic priority for most of such OEMs is to meet short-term financial obligations by exerting their strength to pursue short-term or current gains, instead of increasing resource commitment to unleash their innovation potential for long-term returns in the Chinese market. More specifically, the substantial costs and up-front investment accompanying the innovative activities may outweigh the profits gained from innovativeness and thus deteriorate firm performance. Hence, although many studies show the positive effect of innovativeness on performance, we hypothesize:

#### Hypothesis 1a

In *reverse internationalization*, innovativeness is negatively related to firm performance.

### Risk-taking and performance

Risk-taking that represents a willingness to commit resources to carry out strategies and business activities with significant chances of costly failure is believed to orient SMEs toward a direction of embracing uncertainty as opposed to a fear of it [27]. Despite a positive relationship between risk-taking and performance often found in literature [2], more recent studies have exhibited a negative effect of risk-taking on performance under fast-changing or uncertain circumstances, such as in turbulent times [1] and at the embryonic stage of firm development [20]. Evidence also indicated a concave (inverted U-shaped) relationship between risk-taking and international expansion [23] and a convex (U-shaped) association between risk-taking and SME performance [16], which reveal that lower or higher levels of risk-taking may both lead to the highest level of performance, according to different contexts.

Based on the contradictory findings above, risk-taking may not represent a worthwhile endeavor sometimes, particularly for smaller firms operating in turbulent markets, due to the risk-averse nature of such firms and the substantial resource commitment adhered to risk-taking. Following this logic, given the Chinese global startup OEMs are still striving to neutralize the loss of export revenue in *reverse internationalization* activities, we argue that these firms may bear a relatively low fault tolerance; high levels of risk taking are likely to be too hazardous and counterproductive.

#### Hypothesis 1b

In *reverse internationalization*, risk-taking is negatively related to performance.

### Proactiveness and performance

Proactiveness orients firms towards taking the initiative to position themselves in anticipation of changes, being the first to introduce new offerings in the existing markets, or to enter and develop new markets [27]. Firms with a higher level of proactiveness are able to accommodate to and identify valuable opportunities more quickly than their rivals in a fast-changing context [19].

While carrying out the strategy of reverse internationalization, Chinese global startup OEMs are required to be more sensitive to market signals and better position themselves to obtain a more solid foundation for organizational legitimacy, since they are shifting from competing in well-regulated international arenas to China, an unpredictable, volatile and underdeveloped market [10,22]. As such, *proactiveness* is of greater importance to firms in reacting to “the rules of the games” of unprecedent and vigorous market competition situations.

Proactiveness per se indeed entails a certain levels of innovativeness and risk-taking [7,18]; however, acting proactively is far less risky because *proactiveness* primarily facilitates firms to competing in a more agile manner, and in general won’t result in as much up-front investment as do innovativeness and risk-taking. Hence, we hypothesize:

#### Hypothesis 1c

In *reverse internationalization*, proactivenss is positively associated with firm performance.

### Social network relationships/Guanxi: The moderating effect

According to the literature [17, 20], the controversial and conflicting findings highlight the significance of identifying other factors influencing the EO-performance relationship. The EO-performance linkage has been found to be moderated by a variety of internal and external contingency factors, such as a firm’s capabilities and resources, internal social context, the technological level of the industry, and cultural variables [3, 16]. However, moderators have not yet been sufficiently articulated so far [21], while there remains a scarcity of knowledge about how an entrepreneurial organization’s social networks are leveraged and applied in a specific business circumstance such as *reverse internationalization*. Thus, we intend to examine the moderating effect of a key contingent factor—social networks/*guanxi*- on EO-performance relationships in the Chinese context of reverse internationalization here.

In China where legal and financial institutions are still immature, entrepreneurial firms, in order to achieve competitive advantages, may capitalize on establishing informal social networks with main stakeholders as a critical source of social capital to build legitimacy and credibility in economic exchanges [4, 12, 13, 28]. Such social network relationships may encompass personal and business relations such as buyer-supplier relationships or strategic alliances; nevertheless, here we chose to concentrate on the “social networking”/*guanxi* pertaining to informal structures of personal ties, as it mirrors one salient cultural propensity in China.

Built upon interpersonal trust and goodwill, *Guangxi* as an indigenous Chinese concept with Confucian origins is often characterized by informal interpersonal connections bonded with reciprocal expectations, and has been extended from the individual to the corporate levels [25, 29]. Evidence indicates that *guanxi* provides abundant information benefits and has significant value in enhancing firm performance and entrepreneurial success in the unique institutional environment of China [28]. In general, on the basis of building linkages involved in a business transaction, firms operating in China have to develop and exploit *guanxi* at the organizational level with two major groups of economic actors that provide crucial advantages: 1) Local business network ties: *guanxi* with managers of business stakeholders, e.g., suppliers, buyers, intermediaries and competitors; 2) local institutional network ties: *guanxi* with government officials, key members in trade or industry associations, bankers, and professionals, e.g., professors and scientists [30,31].

*Guanxi* seems to become more critical for Chinese global startup OEMS to harvest from reverse internationalization activities. While these firms are actually latecomers in Chinese market, the development of *guanxi* with local business and institutional partners can provide a buffer for their weak legitimacy, facilitating them to mobilize limited resources in China’s undeveloped, diversified and fragmented regional markets, so as to cope with constraints imposed by highly bureaucratic institutional structures [29]. We thus hypothesize:

#### Hypothesis 2a

In reverse internationalization, *guanxi* moderates the negative relationship between innovativeness and performance; the negative relationship is less pronounced when *guanxi* is better.

#### Hypothesis 2b

In reverse internationalization, *guanxi* moderates the negative relationship between risk-taking and performance; the negative relationship is less pronounced when *guanxi* is better.

#### Hypothesis 2c

In reverse internationalization, *guanxi* moderates the positive relationship between proactiveness and performance; the positive relationship is more pronounced when *guanxi* is better.

### Strategic flexibility: the mediating effect

Scholars point out the imperative to empirically identify more variables intervening in the causal chain between EO and outcomes [21], whereas previous research dealing with the mediation mechanisms of EO-performance relationships mostly focuses on knowledge-based variables, such as organizational learning and knowledge creation process. To fill this gap, here we will unearth the mediating effect of strategic flexibility on EO-performance relationships in China.

*Strategic flexibility* that refers to a firm’s intrinsic ability to leverage and flexibly use resources in reaction to substantial and rapidly occurring environmental changes seems to be a peculiarly effective approach for SMEs to handle institutional constraints embedded in underdeveloped countries. It can impel the entrepreneurial performance of SMEs in unstable and unpredictable emerging economies like China and India, by ushering firms to break down the institutional routines, make precipitate commitments, and undertake competitive actions when facing a variety of changes [32,33]. Strategic flexibility can be seen as an organizational trait as well as one type of *complementary* organizational capability that enables entrepreneurial firms to alter their business practices and strategic initiatives faster than their competitors in terms of grasping new opportunities and tackling treats. In this sense, proactive firms may be more inclined to exhibit strategic flexibility; research has implicitly suggested that *proactiveness* as one of the EO dimensions indeed synthesizes a certain level of such strategic flexibility [1]. Hence, we hypothesize:

#### Hypothesis 3a

Proactiveness is positively related to strategic flexibility.

Some findings highlight the positive effect of strategic flexibility on SME performance during economic downturns because stability may provide a comfort room hindering a firm’s tendency towards entrepreneurship, undermining a firm’s intention to achieve its full potential and flexible usage of key resources [1,33]. In contrast, while entering an emerging and transitional market like our research setting, SMEs may have to perform proactive behaviors to establish business networks, gain access to new niches, and seek out diverse opportunities ahead of their rivals. As a result, strategic flexibility may play a critical role in helping SMEs to continuously depart from their status quo and recalibrate their strategies in a fast-changing environment. Following this logic, it is plausible to postulate that strategic flexibility has a positive impact on firm performance in the context of reverse internationalization.

Integrating the arguments above, we further propose that strategic flexibility is likely to act as a mediator through which Chinese global startup OEMs transfer *proactiveness* into their reverse internationalization performance.

#### Hypothesis 3b

During reverse internationalization, strategic flexibility mediates the positive relationship between EO and performance.

## Methodology

The study was reviewed and approved by an Institutional Review Board at the Institute of Innovation and Development, Hangzhou Dianzi University (ethics committee).

### Sample and data collection

Consistent with Chin and Liu [10], considering the prominent export-oriented nature, “Chinese global startup OEMs” are defined as small-and medium-sized firms in China with export sales accounting for at least 50% of their total sales within 3 years of inception (OEMs refer to the contract manufacturers that are not responsible for the overall value of R & D technology as well as the trademarks of the ultimate products, and anonymous in the final product market). Given that over 50% of FDI into China were found to be pumped into the OEM sector, the number of OEMs has been increasing since the late 90’s; there were more than 5,000 OEMs specializing in shoe production in Guangdong province [34]. As such, to generate a large and representative sample, we relied on the 113 Canton fair exhibitor list to select our sample firms. As a world well-known, comprehensive exhibition held biannually in Guangzhou, this fair has the longest history (55 years) and the largest scale (i.e., the most complete exhibit variety, the broadest distribution of foreign buyers, and the greatest business turnover) in China. More importantly, the participants of this trade fair must supply organizers with clear and accurate demographic and business information about the firm. Hence, in February 2013, we randomly chose firms from the list that fit the definition of Chinese global startup OEMs” above with annual sales of no more than 500 million Yuan (about USD 7.3 million) and fewer than 1,000 employees. To control for the extraneous factor’s influence, the firms with foreign or state-owned shareholders were excluded.

We first made a call to the 300 selected firms and then mailed or emailed a questionnaire to the firms that agreed to join our survey. 21 firms claimed that they have not undertaken reverse internationalization activities and would still focus on export, while 200 firms returned completed questionnaires, yielding a 67% response rate. The informants were chosen from key decision makers, e.g., managing director, chief executive officer, or general manager. To reduce the method bias, we assured the respondents that this is an anonymous survey and there are no right or wrong answers [35]. To validate data, we carried out follow-up telephone interviews shortly after the questionnaires were returned. During this process, we first requested the respondents to indicate their answers to a set of selected questions, and then asked them to recommend a second informant to further confirm the answer. The post survey responses were highly accordant with previous answers.

The final sample included a variety of business domains, e.g., gifts, home decorations, textile garments, electronics, and pharmaceutical products; 24% came from Guangdong province, 14.5% were from Fujian, 23.5% from Zhejian, 8.5% from Guangxi, and 29.5% from other provinces. Referring to the 2002 version of Chinese Standard Industrial Classification, 23.5% of these firms were from technology/capital-intensive industries while 76.5% from labor-intensive industries. The average number of employees per firm is 159 and the average age is 12.1 years. We also conducted a non-response bias test using a within-sample extrapolation method [9]. We compared the early response group (the first 129 responses) and the late response group on key firm characteristics such as firm age, firm size, and industry type. The results of mean comparison t-tests did not return any significant differences between the two groups (p>0.1 in all three characteristics).

### Measures

All items except firms’ information were anchored on a five-point Likert scale (from 1 = “strong disagree” to 5 = “strong agree”; from 1 = a large decrease to 5 = a large increase).

### Domestic performance

Given that entrepreneurs in China experience strong incentives to hide or distort their firms’ financial performance to avoid unwanted attention from corrupt government officials or criminal circles, performance in reverse internationalization was measured by three self-reported indicators (*sales performance*, *market share* and *profitability performance*) referring to previous studies [9,26] (Cronbach α = .942).

### Entrepreneurial orientation

Referring to Covin and Wales [17], “innovativeness” was measured by asking respondents about their firm’s tendency to invest heavily in cutting-edge R & D activities, to be a technological leader, and to introduce innovative products and services during reverse internationalization (3 items, α = .906). Proactiveness was measured by the firm’s propensity to be proactive to change competitive approaches, reorganize operations processes, and to initiate new programs (3 items, α = .800). Risk taking was measured by the firm's preference for taking bold actions and engaging in high-risk projects such as diversifying into new products or service lines, acquiring companies in very different industries, and initiating unknown new business (3 items, α = .740).

### Social network relationships/guanxi

Considering the inherent complexity of the definition of Chinese *Guanxi* and our commercial-oriented backdrop, we merely focused on studying the moderating effect of instrumental and utilitarian *guanxi* (i.e., business and institutional network ties) rather than that of affective, obligatory, or reciprocal *guanxi* (e.g., family, friend, and acquaintance’s relations) [36]. Hence, Referring to Kiss and Danis [31], we measured social networking relationships by asking the respondents to describe how close the links between their firms and the six parties (three on business network ties; three on institutional network ties): (1) key clients; (2) key suppliers; (3) key competitors; (4) governmental officials; (5) key members in trade association or industry policy committee, (6) professionals (e.g., professors, scientists, and bankers) (Cronbach α = .875).

### Strategic flexibility

Consistent with Nadkarni and Herrmann [33], we measured strategic flexibility by asking the respondents to assess the extent to which their firms respond to environmental variations: Our firm (1) regularly shares information with all stakeholders, and (2) frequently changes strategies to derive benefits firm external changes; our strategy (3) emphasizes exploiting new opportunities arising from environmental variability, (4) reflects a high level of flexibility in managing financial and political risks, and (5) emphasizes versatility and empowerment in HR allocation (Cronbach α = .878).

### Control variables

Whereas *firm size*, *firm age*, and *industry type* may influence SMEs’ success [3], we controlled for these variables. *Firm size* was measured as the number of full-time employees; *firm age* as the number of years since it was established. We used a dummy variable to classify the *industry type* into technology and capital-intensive industries (0) or labor-intensive industries (1). In addition, *environmental dynamism* has been found to significantly influence the EO-performance relationship, particularly in a highly dynamic environment riddled with dramatic and rapid changes [5, 12, 37]. Therefore, given the dynamic nature of *reverse internationalization* as noted above, we controlled for environmental dynamism using three indicators according to Wiklund and Shepherd [37]: (1) products have a short life; (2) customers’ demands are highly unpredictable; (3) competitors’ actions are highly unpredictable (Cronbach α = .777).

### Reliability and validity of the measurement model

Cronbach α for all constructs was above 0.70, indicating adequate reliability. We then evaluated the construct validity of the proposed five-factor model by a confirmatory factor analysis (CFA). The CFA results provided a good model fit (χ^2^_n = 200_ = 233.613, df = 119, χ^2^ /df = 1.963<2, p<0.001, CFI = 0.952>0.90, IFI = 0.954>0.90, RMSEA = 0.070<0.08). The assumed five-factor model displayed a good fit to the data, confirming its nomological validity. We further assessed the convergent and discriminant validities by checking for the values of construct reliability (CR) and average variance extracted (AVE). As shown in Table 1, except the second item of the risk-taking scale, all values of factor loadings are higher than 0.5; except risk-taking, all CR and AVE values are above the acceptable levels of 0.6 and 0.5, respectively [9]. One plausible explanation is that during reverse internationalization, global startup OEMs may be unable to bear excessive financial risks like company acquisition (i.e., the second item of the risk-taking scale). Overall, our key measures captured distinct constructs.

**Table 1:** CFA results.

Constructs	Factor loadings	t value	CR	AVE
*Innovativeness*			0.9406	0.8409
Investing heavily in cutting-edge R& D	.909	68.361***		
Tending to be a technological leader	.950	72.689***		
Introducing innovative products and services	.891	65.665***		
*Risk-taking*			0.6531	0.3933
Preferring to undertaking bold actions/high-risk projects	.631	50.162***		
Acquiring companies in different industries	.473	49.551***		
Initiating unknown new business	.747	61.418***		
*Proactivenss*			0.7443	0.5020
Changing the competitive approaches	.822	63.310***		
Reorganizing the operations processes	.691	66.684***		
Initiating specific programs for competing domestically	.594	57.561***		
*Strategic flexibility*			0.8946	0.6335
Regularly sharing information with stakeholders	.828	64.450***		
Frequently changing strategies	.938	61.538***		
Emphasizing on the exploitation of new opportunities	.823	67.339***		
Flexibility in managing financial & political risks	.627	48.007***		
Emphasizing versatility and empowerment in HRM	.729	59.684***		
*Social networking relationship (guanxi)*			0.8574	0.5041
*Guanxi* with key clients	.607	52.499***		
*Guanxi* with key suppliers	.658	56.049***		
*Guanxi* with key competitors	.822	67.525***		
*Guanxi* with governmental officials	.790	73.608***		
*Guanxi* with key members in trade associations	.612	63.944***		
*Guanxi* with professionals	.741	58.985***		

*** Correlation is significant at the 0.001 level.

### Common method bias

Since information about the dependent and independent variables is given by the same respondent, we recognize the potential for common method variance (CMV). Given we had built a five-factor CFA model to examine the construct validity as shown above, we further created a first-order latent marker with all of the measures as indicators to the original CFA model to address the CMV issues. This latent variable approach that has been used in a large body of research allows us to control for the effects of a single unmeasured latent method factor [35]. In comparison with the original five-factor model (χ^2^_n = 200_ = 233.613, df = 119, χ^2^ /df = 1.963, p<0.001, CFI = 0.952, IFI = 0.954, RMSEA = 0.070), the new model with the latent CMV factor still fit the data well (χ^2^_n = 200_ = 225.494, df = 108, χ^2^ /df = 2.088, p<0.001, CFI = 0.951, IFI = 0.953, RMSEA = 0.074) and didn’t make any significant differences (△χ^2^ = 8.119, *p* >.05). We believe that CMV is unlikely to be a major treat here.

## Results and Analysis

### Multiple regression analyses

Scholars have pointed out that examining moderation effects using SEM (i.e., multi-group model) remains extremely difficult [38] and comes with the obvious disadvantage of lower statistical power [39]. As a result, many scholars nowadays still choose to use a hierarchical linear modeling (HLM) to test moderation effects [39–40]. In view of this, we also chose the use of HLM rather than SME to test moderation effects in this paper.

Table 2 presents the means, standard deviations, and correlations of the variables included in our analysis, which confirms that there were no outliers or major violations of regression assumptions. We referred to Cohen et al.’s [41] recommendation to mean-center all independent and moderator variables for minimizing the probability of multicollinearity. The caluculated variance inflation fctors (VIF) were all lower than the cut-off point of 5 [41–42], indicating no apparent multicollinearity.

**Table 2:** Mean, SD, correlations.

	M	SD	1	2	3	4	5	6	7	8	9
1. Firm size	170.63	146.341	1								
2.Industry type	.7650	.42506	-.092	1							
3.Firm age	12.0650	6.08737	.315**	.099	1						
4.Environmental dynamism	-3.5470	.72336	-.060	.044	.049	1					
5.Innovativeness	3.5735	.99490	.056	-.092	-.114	-.415**	1				
6. Risk-taking	3.4821	.73448	-.103	-.053	-.112	-.483**	.394**	1			
7. Proactivenss	3.7320	.70784	.005	-.043	-.197**	-.422**	.555**	.482**	1		
8. Guanxi	3.6012	.67682	.042	-.034	-.091	-.428**	.209**	.398**	.472**	1	
9. Strategic flexibility	3.6802	.65895	.068	-.020	-.211**	-.499**	.549**	.414**	.614**	.413**	1
10. Domestic performance	3.5131	.67913	-.039	-.046	-.333**	-.267**	.156*	.244**	.398**	.401**	.377**

N = 200

**. Correlation is significant at the 0.01 level (2-tailed).

*. Correlation is significant at the 0.05 level (2-tailed).

To assess the explaining power of each set of variables, we followed prior research [1,22] using a HLM with SPSS 21.0 to test the direct influence of each EO dimension on performance and the moderating effect of *guanxi* on the EO-performance relationship. As per the sequential steps of HLM practices, we included all control variables in Model 1A, added independent variables in Model 1B, and added the assumed interation terms in Model 1C. Table 3 presents the summarized results.

**Table 3:** Hierarchical regression analyses.

Variables	Model 1A		Model 1B		Model 1C	
Domestic performance	*ß*	S.E	*ß*	S.E	*ß*	S.E
***Control variables***						
Firm size	0.000	0.000	0.000	0.000	0.000	0.000
Industry type	0.006	0.112	0.010	0.002	-0.039	0.097
Firm age	-0.042***	0.008	-0.037***	0.008	-0.033***	0.008
Environmental dynamism	-0.240***	0.067	-0.086	0.076	-0.039	0.006
***EO dimensions***						
Innovativeness			-0.137*	0.060	-0.167**	0.059
Risk taking			-0.007	0.075	0.053	0.072
Proactiveness			0.356***	0.092	0.363***	0.092
***Moderators***						
Social networking relationships/guanxi			0.227**	0.081	0.218**	0.077
Interactions						
Guanxi* Innovativeness					0.061	0.093
Guanxi* Risk taking					-0.012	0.081
Guanxi* Proactiveness					0.343**	0.131
R^2^	0.189		0.341		0.423	
Adjusted R^2^	0.170		0.311		0.386	
F-value	10.229**		11.145***		11.272***	
△R^2^			0.153***		0.082***	

N = 200

* *p* <0.05

** *p* <0.01

*** *p* <0.001 Significance levels based on two-tailed tests.

Unstandardized regression coefficients are reported; robust standard errors are given in parentheses.

Of the control variables, *environmental dynamism* and *firm age* had negative impact on firm perfrmance as shown in Model 1A(environmental dynamism: *ß* = -0.240, *p* < .001; firm age: *ß* = -0.042, *p* < .001). According to Model 1B and Model 1C, proactiveness had a significant direct positive contribution to firm performance (*p* < .001), while innovativeness showed a signifiacnt negative impact on performance, supporting Hypothesis 1a and 1c. Risk-taking did not have a direct effect on performance, leading to the rejection of Hypothesis 1b.

In Model 1C, the interaction of social networking relationships with proactiveness was significantly and positively related to firm performance (*ß* = 2.185, *p* < .01), supporting Hypothesis 2c. The estimated coefficients of the interactions between guanxi and innovativeness and between *guanxi* and risk-taking were not statistically significant, leading to the rejection of Hypothesis 2a and 2b.

Additionally, Model 1A showed that the control variables explained only 18.9% of the variance in domestic performance. After including three EO variables and *guanxi*, Model 1B explained 34.1% of the variance in performance (△R^2^ = 15.3%, *p* < .001). In Model 1C, the inclusion of interaction terms explained 42.3% of the variance in performance, further adding another 8.2% (*p* < .001) in terms of the explanatory power.

In sum, full support was found for Hypotheses 1a, 1c, and 2c, while Hypotheses 1b, 2a, and 2b were rejected.

### Mediation tests

To test the mediating role of *strategic flexibility* between *proactiveness* and *reverse internationalization performance*, we conducted the structural equation modeling (SEM) approach suggested by Mackinnon et al. [42] to build mediation models. Testing a series of nested models againt the presumed baseline model with sequential chi-sqaure tests enables researchers to better rule out alternative explanations, and so as to discern the model that best illustrates the results of the hypotheses [9, 43]. This approach advocated by Anderson and Gerbing [43] has been widely applied to examine the intervening effects in previous studies [25, 39, 44, 45]. The significance of mediation can be examined by comparing the fit for the direct effect model with that of the predictor-mediator-outcome model (with and without the direct path from the predictor and the outcome constrained to zero) [44, 45]. A full mediation would be identified if the model with the direct path between predictor and outcome displays a better fit.

Table 4 manifests the results of the mediation tests. Consistent with prior studies [25], we estimated a baseline model as the full mediation, which has no direct path from proactivenss to domestic performance. The results of this full mediation model (Model 2A) showed an unsatisfactory model fit (χ2_n = 200_ = 25.690, df = 11, χ^2^/df = 2.335>2, *p<*0.01; CFI = 0.934>0.90; NFI = 0.897<0.90; IFI = 0.939>0.90; RMSEA = 0.082>0.08). Then we tested a series of nested models against our baseline one via sequential chi-square tests with the parameter constraints of interest in our research. We comapred Model 2A with a partial mediation model (Model 2B) in which the path from proactiveness to performance was added. As seen in Table 4, the fit indices of Model 2B look more acceptable than that of the baseline model (χ2_n = 200_ = 9.345, df = 9, χ^2^/df = 1.038<2, p = 0.406>0.05; CFI = 0.998>0.90; NFI = 0.963>0.90; IFI = 0.999>0.90; RMSEA = 0.014<0.05), considering the significant chi-square difference between Model 2A and Model 2B (△ χ^2^ = 16.345, df = 2, *p*<0.05).

**Table 4:** Hypothesis test of alternative models.

	χ^2^	Df	χ2*	df*	IFI	NFI	CFI	RMSEA
Baseline Model 2A	25.690	11	-	-	0.939	0.897	0.934	0.082
Model 2B	9.345	9	9.027	2	0.999	0.963	0.998	0.014
Model 2C	68.218	10	42.528	1	0.757	0.727	0.738	0.177
Model 2D	37.791	10	12.101	1	0.884	0.849	0.875	0.118
Model 2E	28.998	10	3.308	1	0.921	0.884	0.914	0.098

N = 113.

*. The differences between the model and the basic model 2A.

Baseline model: full mediation (no direct paths from the predictor to outcome).

Model 2B: partial mediation model (the path from the predictor to outcome was added).

Model 2C: direct effects model (the path from the predictor to mediator was constrained to zero).

Models 2D: non-mediation models (the path from the mediator to outcome was constrained to zero).

Models 2E: reverse causality models: Strategic flexibility to proactiveness to domestic performance.

Model 2C is a direct effect model, where there is no causal relationship between the proactiveness and strategic flexibility, as proactiveness and strategic flexibilty were set to directly link performance. Model 2D is a non-mediation model assuming that the path from strategic flexibility to performance was constrained to zero. Model 2E represents a reverse causality model that treats *strategic flexibility* as an antecedent of proactiveness, whereby proactiveness conversely mediated the relationship between strategic flexibility and performance. According to Table 4, the fit indices of Model 2B are significantly superior to those of Models 2A, 2C, 2D, and 2E. Overall, the results suggest that the partial mediation model 2B best fit our data.

### Assessment of hypotheses

**Fig 1:**
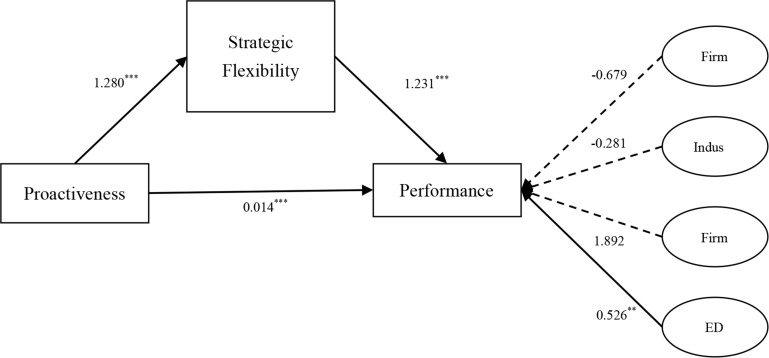
The Mediation Model.

Fig 1 delineates the parameter estimates of the final best fit model 2B for examining our hypotheses 3a and 3b. Hypothesis 3a states that proactiveness is positively related to strategic flexibility. As shown in Fig 1, the direct path from proactivenss to domestic strategic flexibility is significant (*β* = 1.280, *p* <0.001), supporting Hypothesis 3a. Hypothesis 3b assumes that strategic flexibility mediates the effect of proactiveness on domestic performance. According to Fig 1, the direct path from proactiveness to performance is still significant (*β* = 0.014, *p*<0.001) while the indirect path from proactiveness via strategic flexibility to performance is significant (from proactiveness to strategic flexibility: *β* = 0.1280, *p* <0.001; from strategic flexibility to performance: *β* = 0.1231, *p* <0.001). Hypothesis 3b is strongly supported as well.

## Discussion

This present research illustrates that during China’s economic transition period, the link between EO and firm performance presents more complicated interrelationships than a simple direct relationship, and the individual dimensions of EO may have different influences on firm performance. As per our findings, in the context where global startup OEMs return to compete domestically, **proactiveness** is positively related to firm performance; **risk-taking** is not statistically associated with performance; **innovativeness** is negatively related to performance. In alignment with recent findings [16, 23], our results confirm that three dimensions of EO demonstrate differential associations with SME performance. More specifically, in the context of reverse internationalization by Chinese global startup OEMs, proactiveness that is generally consider a cornerstone of the role EO plays in driving firm performance [1,7] indeed exhibits exclusively positive effects.

In contrast, during reverse internationalization, innovativeness that entails lots of R & D cost may impede performance, whereas risk-taking that deals with large possibilities of failure has no significant impact performance in the given context. SMEs with higher level of innovativeness are believed to be beneficial in dynamic emerging markets [10]. However, based on our findings, entrepreneurial OEMs operating on thin profit margins may not be able to rely on undertaking innovative projects to increase revenue in China, owing to the fact that a variety of institutional barricades and less intellectual property protection can diminish the value of innovativeness [24]. Kraus et al. [1] also suggest that in the face of an economic slowdown, SME with severe financial pressure will need to have a legacy of innovativeness to draw upon when entering new markets as opposed to building it directly from new markets. As such, it seems plausible that Chinese global startup OEMs may typically not capitalize on innovativeness to generate competitive advantages in the context of reverse internationalization, since they used to prefer imitation and low-cost manufacturing rather than innovation and R & D input, thus remaining weak in the initiation of new technologies [9].

The contradictory findings about the impact of risk-taking on performance have led to an increasing level of scrutiny in recent entrepreneurial literature [16]. A large body of studies has manifested that risk-taking behaviors often don’t represent a worthwhile entrepreneurial endeavor for SMEs but display a predominantly direct or U-shaped negative relationship with firm performance [23]. Consistent with Krasus et al.[1], risk-taking does not show a direct association with performance in our research. However, it may be caused by that the second indicator of our risk-taking scale, as noted above, seems not suitable for our background setting of *reverse internationalization* because Chinese global startup OEMs with heavy financial burden are unlikely to view the acquisition of new business as their strategic priority.

Our study demonstrates that social networking relationship (*guanxi*) significantly moderates the effect of proactiveness on performance, while the interactions of *guanxi* with risk-taking and innovativeness have no significant impact on performance. Implicitly in the findings corroborate the vital role of social networking with two dominant resource controllers- the key persons of local government agencies and business communities- in facilitating SMEs’ awareness and access to valuable market information in China [19, 22, 29]. With *guanxi* as a precious information source, it would be easier for proactive firms to foresee future trends of the Chinese markets; it is understood why the positive effects of proactiveness on performance becomes stronger when interacting with *guanxi*.

In terms of the mediating effect, our results present that proactivenss can be translated into firm performance through strategic flexibility. As is well-known, China’s socialist political structure allows regional governments to be directly involved in the enactment of market policies in their jurisdictions, and thus the market structures vary from region to region [34, 46]. Due to such complicated political embeddedness in the Chinese market with the corresponding decentralized and segmented market design [4, 46, 47], merely engaging in proactive behaviors could be ineffective if entrepreneurial firms are unable to compensate for their resource shortages. Hence, strategic flexibility embodying the firm’s ability to coordinate and leverage limited resources appears to be particularly instrumental for SMEs to transform their entrepreneurial efforts such as adjusting competitive approaches and reallocating resources into actual performance in China. In other words, without a certain degree of strategic flexibility, Chinese global startup OEMs, despite possessing a high level of proactivenss, may still not be able to achieve superior performance when undertaking reverse internationalization.

It is worth noting that *firm age* as a control variable elicits negative impact on firm performance according to our results. Our findings to a certain extent support the theory of learning advantages of newness (LAN) [48], which assumes that the latecomers may not suffer from the same inertial forces that stifle mature incumbents’ adaptation to new changes in target markets. In this regard, the global startup OEMs, as newcomers in their domestic competitions, might also derive competitive advantages from accelerated cross-border learning.

Overall, several contributions emerge from this current research. First, we explore how the individual dimensions of EO benefit, suppress or have no significant effect on firm performance in a particular environment, in response to the recent calls for unraveling the heterogeneous characteristics of each sub-dimension of EO as well as for more contextually embedded treatment of the EO-performance association [6,8]. Second, by testing the moderating role of *guanxi* and the mediating mechanism of strategic flexibility on EO-performance relationships, we advance the understanding of how such contingent factors facilitate entrepreneurial OEMs to break down institutional barriers and reap the most from EO in the transitional economy of China. Third, we contribute to the literature by introducing and elucidating a novel entrepreneurial phenomenon, ‘reverse internationalization’ by Chinese global startup OEMs. Although China’s tremendous domestic market and huge consumption demand have become a major source of growth for world economy, such entrepreneurial activity has had limited examination in the literature [9, 24]. This paper, therefore, delivers a clearer, holistic picture of this intriguing entrepreneurial scene in the Chinese market, providing valuable and practical implications for other OEMs in China to deal with relevant issues in a better manner.

## Limitations and Future Research

This paper, as any other research, has its boundaries and limitations, which may open up fertile avenues for future research in relevant fields. First, using cross-sectional data may not be sufficient for establishing proper causality between EO and performance [13]; future research could conduct a longitudinal study to interpret the key issues more comprehensively and precisely. Second, employing perceived and aggregated performance index may mask the respective effects of EO on individual financial indicators, nonfinancial performance, and growth potential [1]. Future research should take in to consideration the utilization of more solid financial performance indices such as archival information and the inclusion of various types of performance measures (e.g., customer satisfaction). Third, this research is limited in data collection. Future research is encouraged enlarge the sample size, incorporate multiple informants, and add more diverse control variables for a deeper understanding of relevant issues. Fourth, though most scales of social networking relationships/guanxi assess only the perceived value of *guanxi*, future studies should consider the development of more objective measurement to capture the quality of the business and institutional network ties. Additionally, given that the EO-performance relationship is bounded to contextual contingencies [23, 27], it is vital to further investigate under what circumstances SMEs with high EO can outperform competitors and how the sub-dimensions of EO interact with other idiosyncratic factors to affect performance. Doing so will provide valuable reference points for future generations of EO researchers.

In conclusion, the fast-changing and complex Chinese market, coupled with the tough issues of overcapacity and rising labor cost in China’s OEM industry, might be able to best elaborate on why our distinctive results occurs. This current research-which brings new insights into how Chinese global startup OEMs benefit from EO in the process of reverse internationalization- unveils a distinctive EO-performance mechanism in a given context that is quite different than that in mature and developed markets.

## Supporting Information

S1 File Questionnaire Information (DOC)
