# Supplementary material for: EO-Performance relationships in Reverse Internationalization by Chinese Global Startup OEMs: Social Networks and Strategic Flexibility
Source: PLoS One. 2016 Sep 15;11(9):e0162175. doi: 10.1371/journal.pone.0162175 (PMC5025104; doi:10.1371/journal.pone.0162175)
Supplement: S1 File — (DOC) [file pone.0162175.s001.doc]

**Questionnaire information**

| **Firm Information:** |  |  |  |  |  |
| --- | --- | --- | --- | --- | --- |
| Firm size (Number of Full-time Employees) : |  |  | Industry type: | |  |
| Firm age (number of years since established) : |  |  | Firm location: | |  |
|  |  |  |  |  |  |
| **Questions** | **Strongly Disagree** | **Disagree** | **Undecided** | **Agree** | **Strongly Agree** |
| **Innovativeness** |  |  |  |  |  |
| Investing heavily in cutting-edge R& D | 1 | 2 | 3 | 4 | 5 |
| Tending to be a technological leader | 1 | 2 | 3 | 4 | 5 |
| Introducing innovative products and services | 1 | 2 | 3 | 4 | 5 |
| **Risk-taking** |  |  |  |  |  |
| Preferring to undertaking bold actions/high-risk projects | 1 | 2 | 3 | 4 | 5 |
| Acquiring companies in different industries | 1 | 2 | 3 | 4 | 5 |
| Initiating unknown new business | 1 | 2 | 3 | 4 | 5 |
| **Proactivenss** |  |  |  |  |  |
| Changing the competitive approaches | 1 | 2 | 3 | 4 | 5 |
| Reorganizing the operations processes | 1 | 2 | 3 | 4 | 5 |
| Initiating specific programs for competing domestically | 1 | 2 | 3 | 4 | 5 |
| **Strategic flexibility** |  |  |  |  |  |
| Regularly sharing information with stakeholders | 1 | 2 | 3 | 4 | 5 |
| Frequently changing strategies | 1 | 2 | 3 | 4 | 5 |
| Emphasizing on the exploitation of new opportunities | 1 | 2 | 3 | 4 | 5 |
| Flexibility in managing financial & political risks | 1 | 2 | 3 | 4 | 5 |
| Emphasizing versatility and empowerment in HRM | 1 | 2 | 3 | 4 | 5 |
| **Social networking relationship (guanxi)** |  |  |  |  |  |
| Guanxi with key clients | 1 | 2 | 3 | 4 | 5 |
| Guanxi with key suppliers | 1 | 2 | 3 | 4 | 5 |
| Guanxi with key competitors | 1 | 2 | 3 | 4 | 5 |
| Guanxi with governmental officials | 1 | 2 | 3 | 4 | 5 |
| Guanxi with key members in trade associations | 1 | 2 | 3 | 4 | 5 |
| Guanxi with professionals | 1 | 2 | 3 | 4 | 5 |
| **Environmental dynamism** |  |  |  |  |  |
| Products have a short life | 1 | 2 | 3 | 4 | 5 |
| Customers’ demands are highly unpredictable | 1 | 2 | 3 | 4 | 5 |
| Competitors’ actions are highly unpredictable | 1 | 2 | 3 | 4 | 5 |

问卷题项

| **企业信息** |  |  |  |  |  |
| --- | --- | --- | --- | --- | --- |
| 企业规模（全职员工人数）： |  |  | 企业所处产  业类型： | |  |
| 企业年龄： |  |  | 企业所处国  家与省份： | |  |
|  |  |  |  |  |  |
| **问题** | **非常不同意** | **不同意** | **不确定** | **同意** | **非常同意** |
| **创新性** |  |  |  |  |  |
| 企业趋向于大力投资于尖端技术或产品的研发 | 1 | 2 | 3 | 4 | 5 |
| 企业趋向于成为一个行业技术领导企业 | 1 | 2 | 3 | 4 | 5 |
| 企业趋向于推出创新的产品与服务 | 1 | 2 | 3 | 4 | 5 |
| **风险承受** |  |  |  |  |  |
| 企业勇于参加高风险项目 | 1 | 2 | 3 | 4 | 5 |
| 企业勇于收购不同行业的公司 | 1 | 2 | 3 | 4 | 5 |
| 企业勇于开展具有不确定性的新业务 | 1 | 2 | 3 | 4 | 5 |
| **主动性** |  |  |  |  |  |
| 企业倾向于主动改变竞争方式 | 1 | 2 | 3 | 4 | 5 |
| 企业倾向于主动重组运作流程 | 1 | 2 | 3 | 4 | 5 |
| 企业倾向于主动为国内竞争开拓特色项目 | 1 | 2 | 3 | 4 | 5 |
| **战略灵活性** |  |  |  |  |  |
| 企业经常与利益相关者分享信息 | 1 | 2 | 3 | 4 | 5 |
| 企业频繁地改变战略 | 1 | 2 | 3 | 4 | 5 |
| 企业强调开拓新的机会 | 1 | 2 | 3 | 4 | 5 |
| 企业灵活地管理金融与政治风险 | 1 | 2 | 3 | 4 | 5 |
| 企业在人力资源管理中强调多功能性与权利下放 | 1 | 2 | 3 | 4 | 5 |
| **关系** |  |  |  |  |  |
| 企业与主要客户的关系 | 1 | 2 | 3 | 4 | 5 |
| 企业与主要供应商的关系 | 1 | 2 | 3 | 4 | 5 |
| 企业与主要竞争者的关系 | 1 | 2 | 3 | 4 | 5 |
| 企业与政府官员的关系 | 1 | 2 | 3 | 4 | 5 |
| 企业与贸易商会主要成员的关系 | 1 | 2 | 3 | 4 | 5 |
| 企业与专家的关系 | 1 | 2 | 3 | 4 | 5 |
| **环境动态** |  |  |  |  |  |
| 企业的产品具有较短生命周期 | 1 | 2 | 3 | 4 | 5 |
| 企业的客户需求具有非常高的不确定性 | 1 | 2 | 3 | 4 | 5 |
| 企业的竞争对手的行为具有非常高的不确定性 | 1 | 2 | 3 | 4 | 5 |
